# Dimensional stability and surface hardness of inter-occlusal recording materials

**DOI:** 10.6026/973206300200344

**Published:** 2024-04-30

**Authors:** Khyati N. Gosai, Ina B. Patel, Pooja Poonia, Bhagyashree Sutaria, Mahima Trivedi, Sarita Mori

**Affiliations:** 1 Department of Prosthodontics Crown and Bridge, Maharaja Ganga Singh Dental College and Research Centre, Sri Ganganagar, Rajasthan, India; 2 Department of Prosthodontics Crown and Bridge, AMC Dental College and Hospital, Ahmedabad, Gujarat, India; 3 Department of Prosthodontics Crown and Bridge, Ahmedabad Dental College, Ahmedabad, Gujarat, India; 4 Department of Prosthodontics Crown and Bridge, Siddhpur Dental College and Hospital, Gujarat, India; 5 Dental Studio, Vadodara, Gujarat, India; 6 Department of Periodontology, Ahmedabad Dental College, Ahmedabad, Gujarat, India

**Keywords:** Inter-occlusal registration materials, bisacrylate luxabite, polyvinyl siloxane, aluwax

## Abstract

It is important to choose the appropriate interocclusal registration material for precise articulation and successful dental prosthesis fabrication. 3 types of interocclusal registration materials: Bite registration wax, polyvinyl siloxane bite registration paste and Bisacryl-based bite registration paste were evaluated for dimensional stability and surface hardness at 4 different time intervals. One way ANOVA test, multiple Post Hoc Tukey HSD test and nonparametric test were performed. Bisacryl-based bite registration material exhibited better dimensional stability and surface hardness than polyvinyl siloxane and bite registration wax at all 4 time intervals. Thus dimensional stability and surface hardness of interocclusal registration material was influenced by both the type of material and the time duration.

## Background:

Accurate Prosthodontic rehabilitation relies on methods and materials used to record occlusion and precise articulation of the patient's casts requires recording the existing maxillo-mandibular relationships with the help of proper interocclusal recording materials in tripodal contacts [1]. The horizontal stability is essential to prevent the horizontal rotation between the casts and is generally obtained using interocclusal registration material [1]. Thus, it is important to use dimensionally stable interocclusal recording materials to accurately represent the patient's maxillo-mandibular relationship [2]. In earlier times, various materials like impression plaster, impression compound, modeling wax, ZOE paste, eugenol-free zinc oxide paste, and acrylic resin were routinely used for the registration of occlusal relationships [3]. Recently Elastomeric materials and modified resins are also available [3]. According to Schnader [4], ZOE is brittle, dimensionally unstable, and cannot be reused for articulation. The drawbacks of Modelling wax are inaccuracy and distortion. Martin H. Berman [5] recommended the use of Alumina filler in wax (Aluwax) to improve dimensional stability. Auto Polymerizing Acrylic resin undergoes polymerization shrinkage and thus is dimensionally unstable [6]. PVS interocclusal recording material is widely used as interocclusal recording material due to its ease of availability, manipulation, less setting time, accuracy in surface reproduction, dimensional stability, good compressive strength, and hydrophobic nature [7]. Bisacrylates are resins with glass fillers and reduced amounts of methyl methacrylate and peroxides compared to traditional resins making them rigid, resistant to temperature variation, not spongy as PVS material and polyether, and are dimensionally stable . Thus, the interocclusal registration material should be easy to [7] manipulate, cost-effective, dimensionally stable, resistant to deformation on storage, resistant to deformation on the application of compressive load during articulation, and have adequate surface hardness. Hard and highly filled inter-occlusal recording materials ensure more accurate fit on stone models [8]. Therefore, it is of interest to compare the time-dependent linear dimensional stability and surface hardness of conventional and newer interocclusal registration materials.

## Materials and Method:

An in-vitro study was conducted in the Department of Prosthodontics and Crown & Bridge, AMC Dental College and Hospital, Ahmedabad, Gujarat under permission from the Institutional Review Board (IRB) and the microscopic evaluation was performed at the Central Institute of Plastic Engineering and Technology (CIPET), Testing Department, Ahmedabad, Gujarat. Interocclusal recording materials used are Alu wax (Maarc), polyvinyl siloxane (Dental Avenue-Avue bite) and Bisacryl Luxabite (DMG-Luxabite). Sample size of 30 was selected, 10 specimens for each material(group A, group B, and group C) with 3 mm thickness, visible lines, even color, and no voids or cracks were included in the study. Specimens not following these criteria were excluded. The armamentarium used for the study were Stainless steel die (ADA specification- 19), Thermostat control water bath unit, Auto-mixing gun with mixing tips, Polyethylene sheet, 500 g weight, Bard Parker blade no 15 and Digital microscope- 10 X magnification. Stainless steel die was prepared according to revised ADA specification no.19 for non-aqueous elastic dental impression materials [9]. It consisted of a ruled block (AA) and test material mold (BB). The ruled block had three horizontal lines of different widths; a small Y-line (24 µm), a medium X-line (57 µm), and a thick Z-line (83µm), and two vertical lines CD and CI DI of 82 µm each. The lines CD and CI DI were separated by 25 mm approx. The test mold had a cylinder with an inner diameter of 30 mm and depth of 6 mm to place the bite registration material which acted as mold space for the material (Figure 1 - see PDF). The die was subjected to Nd-YAG Laser treatment and 3 horizontal, 2 vertical lines were scribed, with a width of 0.016 mm on top of a 30 mm diameter surface. The die also contains a ring that surrounds the periphery of it, which serves as a tray or as a container for the interocclusal recording material.

## Manipulation of bite registration material and preparation of test specimens:

Bite registration wax (Aluwax) was broken and inserted into a 5 ml glass syringe, submerged then in a 45°C water bath for 5 minutes and melted wax was poured into the die. The PVS bite registration paste and luxabite available in the form of base and accelerator paste were expelled from the auto-mixing gun and uniformly spread over the surface of the die. 10 samples for each material were made. A 4X4 glass plate covered with a polyethylene sheet was placed on the die. Hand pressure was applied for 5 seconds initially to expel the material, over which a weight of 500 gms was kept to simulate biting pressure. The whole assembly was then submerged in a thermostatically controlled water bath at a temperature of 36±1°C resembling open mouth temperature for the time suggested by the manufacturer, plus an additional 3 minutes. Thus prepared specimens measured 30 mm in diameter and 3 mm in height. All specimens had lines X, Y, Z, and CD and CI DI lines on them (Figure 2 - see PDF).

## Evaluation of dimensional stability:

The distance between the lines, CD and CI DI, was reproduced on the samples and it was measured at three different points PPI, QQI, and RRI (i.e. at the intersections of these lines with the lines XYZ) by using digital microscope with 10 X magnification (Figure 3 - see PDF). Three readings were received for every fabricated sample and the averages of those 3 values were noted. Likewise, readings were made at different time intervals i.e.; 1 hour after removal of the material from the die, at 24 hours, at 48 hours, and at 72 hours respectively for each of the samples.

## Evaluation of dimensional change and surface hardness:

Dimensional error was calculated by the mean distance between lines -PPI, QQI, and RRI (mm). Materials having the least mean dimensional error were most dimensionally stable. Surface hardness was evaluated after 24 hours using Shore hardness tester machine. The pointed needle of the instrument was inserted into the sample and readings were obtained accordingly. (Figure 4 - see PDF)

## Results:

For dimensional stability of all three interocclusal recording materials- Aluwax, Polyvinylsiloxane and Bisacryl Luxabite at 1 hour, 24 hours, 48 hours and 72 hours -Mean, Mean dimensional error and standard deviation was calculated. The test performed were one way ANOVA Test for quantitative data with multiple Post Hoc Tukey HSD Test and categorical data using a nonparametric test- Repeated Measures ANOVA Test using SPSS Statistics software v23; IBM Corp. For all statistical analyses, probability levels of P < 0.05 were considered statistically significant.ANOVA test was performed for the dimensional stability of 3 interocclusal recording materials after 1 hour, 24 hours, 48 hours and 72 hours which showed P value < 0.0001 thus indicating statistical significant difference in the dimensional stability of all 3 materials (Figure 5 - see PDF).

Post Hoc Tukey HSD Test for intergroup comparison 1 hour, 24 hours, 48 hours and 72 hours after removing from test apparatus resulted in Statistically significant difference (p<0.05) between Alu wax v/s Polyvinylsiloxane and Alu wax v/s Bisacryl luxabite except Polyvinylsiloxane v/s Bisacryl luxabite which showed insignificant difference where P value is 0.317 (P>0.05) at 1 hour, P= 0.457 (P>0.05) at 24 hours, P= 0.406 (P>0.05) at 48 hours. Statistically significant difference (p<0.05) was seen for Polyvinylsiloxane v/s Bisacryl-Luxabite at 72 hours.

Dimensional change in each material - Aluwax, Polyvinylsiloxane and Bisacryl Luxabite at each time interval- 1hour, 24 hours, 48 hours and 72 hours was statistically analysed by the means of repeated measures ANOVA and it showed P value < 0.0001 thus indicating statistical significant difference in the dimensional stability of all 3 materials. For surface hardness of all three interocclusal recording materials were tested at 24 hours. Mean and Standard Deviation was calculated (Figure 6 - see PDF). The test performed was ANOVA Test and Post Hoc Tukey HSD Test.

ANOVA test was performed for the surface hardness of 3 interocclusal recording materials after 24 hours which showed P value < 0.0001 thus indicating statistical significant difference in the surface hardness of all 3 materials. Post Hoc Tukey HSD Test for intergroup comparison of hardness was performed and Statistically significant difference (p<0.05) was seen between all the groups of bite registration material- AluWax v/s Polyvinylsiloxane, AluWax v/s Bisacryl-Luxabite and Polyvinylsiloxane v/s Bisacryl-Luxabite.

## Discussion:

An accurate transfer of the interocclusal relationship to the articulator is essential for a prosthesis fabrication. In this in-vitro study, the time-dependent linear dimensional stability and surface hardness of three interocclusal registration materials were measured. The time intervals were based on the time required to carry the interocclusal registration material to a distant laboratory leading to the delay in the articulation of the cast. An increased value of dimensional error shows lesser dimensional stability. Also, more surface hardness of the bite registration material provides accurate articulation of the cast. Results showed that the changes in dimensional stability were comparatively insignificant for Bisacryl Luxabite at all 4-time intervals but significant changes were found in PVS bite registration material and Aluwax at all 4 time intervals. Also, the linear dimensional change in each material started from a time interval of 1 hour. So it was concluded that interocclusal recording material should be used within 1 hour for best results for articulation. According to this study, linear dimensional changes were statistically significant for PVS interocclusal registration material. The results agreed with studies done by Michalakis *et al.* [10], which showed a dimensional change of 0.18% after 24 hours and it kept on increasing till 168 hours and Ghazal *et al.* [11], where dimensional error of PVS material at 48 hours was more than luxabite. Tejo *et al.* [12] also concluded that dimensional changes were seen after 72 hours in PVS material which was more as compared to Polyether material. Gaurav *et al.* [13] showed that PVS material displayed significant dimensional change at 24 hours continuing till 8 days. Mithra *et al.* [14] showed that PVS record material showed a significant dimensional change up to 48 hours, after that it was stable. Megremis *et al.* [15] concluded that PVS material showed a linear dimensional change of 0.5 percent or less till 72 hours. Several factors like loss of volatile substance i.e., hydrogen gas contributed to the dimensional changes of the elastomeric materials due to polymerization shrinkage. This finding was confirmed by a study conducted by Myerson *et al.* [16] which stated that there is a correlation between volatile substance loss-induced weight loss and linear changes in only the horizontal plane in elastomeric interocclusal recording materials. However, the result of this study was contradicted by Muller *et al.* [17] who reported that PVS interocclusal records can be articulated only upto 24 hrs as they show dimensional changes after 24 hours and wax records must be articulated within 1 hour to get accurate restoration. Also, Gupta *et al.* [18] concluded that PVS material was the most dimensionally stable bite material with high surface hardness at the time interval of 24 hours. A study done by Dua *et al.*[19] and Philip Millstein *et al.* [20] concluded that insignificant dimensional error were seen in PVS material even after 24 hours and 48 hours respectively. Arya *et al.* [21] concluded that the dimensional stability of the PVS interocclusal record was highest from 1 hour to 168 hours as compared to Luxabite. The results of this study showed maximum dimensional changes in Aluwax. This result was per a study done by Lassila *et al.* [22] which concluded that wax is unreliable as interocclusal registration material because of its considerable cooling contraction and high thermal coefficient. Mullick *et al.* [23] concluded that wax interocclusal records were the least reliable for articulation as they showed maximum dimensional errors. Various tests can be performed to measure surface hardness such as Barcol, Brinell, Rockwell, Vickers, Knoop, and Shore, where shore hardness test has been used for measuring the hardness of rubber and plastic types of dental materials [24]. Hence in this study, the hardness of Aluwax and elastomeric interocclusal recording material was checked by shore hardness test using a shore hardness tester instrument at 24-hours intervals. Only a few studies are performed in the literature for comparing the surface hardness of Polyvinylsiloxane and Bisacryl-based luxabite.

## Conclusion:

Surface hardness and dimensional stability was highest for Bisacryl-Luxabite may be due to the presence of more filler and glass matrix along with less amount of methyl methacrylate and peroxides which generally contribute to less shrinkage during setting. Alu wax showed dimensional changes with time because of thermal changes as it has high thermal coefficient and for PVS material dimensional changes occur due to cross-linking during polymerization or may be due to loss of volatile substances along with its spring back mechanism due to rubber base. Within the limitations of this study, it is concluded that Bisacryl-based Luxabite is superior in both properties as compared to the other two materials so can be used upto 72 hours of articulation, but when unavailable polyvinylsiloxane and least preferably aluwax can be a choice but should be used immediately.

## List of Abbreviations:

PVS: poly vinyl siloxane

ZOE: Zinc oxide eugenol

ADA: American dental association

SD: Standard Deviation
